# A structural overview of GH61 proteins – fungal cellulose degrading polysaccharide monooxygenases

**DOI:** 10.5936/csbj.201209019

**Published:** 2012-11-30

**Authors:** Leila Lo Leggio, Ditte Welner, Leonardo De Maria

**Affiliations:** a Department of Chemistry, University of Copenhagen, Universitetsparken 5, 2100, Copenhagen Ø, Denmark; b Statens Seruminstitut, Artillerivej 5, 2300 Copenhagen S, Denmark; c Novozymes A/S, Krogshøjvej 36, 2880 Bagsvaerd, Denmark

## Abstract

Recent years have witnessed a spurt of activities in the elucidation of the molecular function of a class of proteins with great potential in biomass degradation. GH61 proteins are of fungal origin and were originally classified in family 61 of the glycoside hydrolases. From the beginning they were strongly suspected to be involved in cellulose degradation because of their expression profiles, despite very low detectable endoglucanase activities. A major breakthrough came from structure determination of the first members, establishing the presence of a divalent metal binding site and a similarity to bacterial proteins involved in chitin degradation. A second breakthrough came from the identification of cellulase boosting activity dependent on the integrity of the metal binding site. Finally very recently GH61 proteins were demonstrated to oxidatively cleave crystalline cellulose in a Cu and reductant dependant manner. This mini-review in particular focuses on the contribution that structure elucidation has made in the understanding of GH61 molecular function and reviews the currently known structures and the challenges remaining ahead for exploiting this new class of enzymes to the full.

## Introduction

Decades of research on plant polysaccharide degrading enzymes for the exploitation of biomass have mostly focused on glycoside hydrolases, which have been classified in sequence-based families in the CAZY (Carbohydrate Active enZYmes) database [1]. Glycoside hydrolases (GH) and other carbohydrate active catalytic domains are often coupled to non-catalytic carbohydrate binding modules (CBMs, reviewed in [2]), also classified in CAZY, which have the function of binding to *eg* crystalline or complex substrates and have in some cases been shown to act in synergy with the catalytic domains.

In view of the world energy crisis, bioethanol production has become a rather hot topic. While ethanol can be feasibly produced from starch rich crops, a much more renewable and sustainable solution would be the production from (ligno)cellulosic biomass, which constitutes a large proportion of agricultural and forestry byproducts. Thus a lot of attention has been devoted to enzymes able to degrade cellulose to sugars fermentable by *S. cerevisiae*. In particular the *Trichoderma reesei*/*Hypocrea jecorina* system has received much attention in terms of commercial exploitation. Cellulose breakdown (see [3] for a classic review) has been viewed for many years as carried out mainly by endoglucanases and processive exoglucanases (cellobiohydrolases) acting in synergy, often with the aid of cellulose binding domains assisting attachment to cellulose. β-glucosidases are also often part of cellulolytic systems, where they relieve the product inhibition of cellobiohydrolases by cellobiose, and they are often added to commercial preparations. However, the mechanism by which some microorganisms are able to efficiently degrade crystalline cellulose has remained in many ways a mystery. In the last few years a new class of fungal proteins with huge potential for the degradation of cellulose has received much attention, the GH61 proteins. Initially classified as family 61 among the glycoside hydrolases they are now recognized to be Cu-dependent oxidases [4–6], calling for a reclassification of these enzymes. As such a reclassification is yet to be implemented in the CAZY database, we choose in this review to keep the somewhat inappropriate GH61 designation, which allows retrieval of most of the earlier literature. This family has puzzled carbohydrate active enzyme experts since its discovery, and to some extent continues to do so. Structure determination by X-ray crystallography was a crucial step towards understanding the significance and mechanism of action of these enzymes. This short review briefly summarizes the progress up to now, and focuses on the structures currently known.

## A brief history of GH61

**Figure 1:**
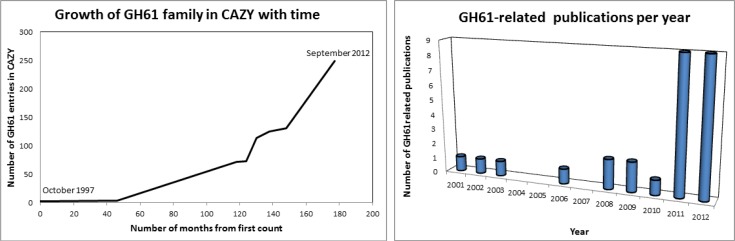
A) Number of GH61 members in CAZY from October 1997, when the new family was first announced in publications [8, 9]. The first count after the family was formed was in August 2001 according to [10]. Subsequent counts were made with the help of the Wayback Machine (http://archive.org/web/web.php). B) Number of articles in Pubmed with ‘GH61’ or 'family 61’ in title/abstract for each year (checked for relevance). Some publications which include information on GH61 may not be included in the count, if they did not include the chosen search keywords in abstract or title.

The first GH61 protein to be identified was probably CelI from *Agaricus bisporus* the sequence of which was described in 1992 after cloning of the gene [7]. Although no activity could be described, the gene was induced on growth on cellulose, and the presence of a sequence typical of a cellulose binding domain implicated the protein in cellulose degradation. The GH61 family was first created in 1997, when it was referred to at least twice in the literature [8, 9]. The evolution of the family in terms of number of members can be seen in Figure 1a.

The first papers on characterization of GH61 family members reported very low cellulose degrading activity if any. For example *T. reesei* Cel61A [10], showed some degrading activity on polymeric cellulosic substrates, but at levels 5-6 orders of magnitude lower than a conventional cellulase, Cel7B, making it difficult even by use of sensible controls to totally rule out the possibility of contamination by canonical cellulases. In hindsight, the low activity can be explained by the lack of essential cofactors, which at the time were unknown. However the identification of GH61 members in cellulolytic organisms such as *T. reesei*, *A. bisporus*, *Aspergilli* species and *Neurospora crassa* together with their co-induction with classical cellulases upon growth on cellulose [7, 11], already early on suggested the GH61 family involvement in lignocellulose degradation. This was further supported by the fact that several of the first GH61 domains were found to be associated with family 1 CBMs, which are crystalline cellulose binders.

A first breakthrough in the molecular understanding of GH61 function came from the structures of two family members which were communicated at conferences and in peer-reviewed journals in 2008 [12, 13]. The first publication [13] revealed the 3D structure of *Hypocrea jecorina* (*Trichoderma reesei*) Cel61B (from now on referred to as HjGH61B), solved by Single-wavelength Anomalous Diffraction utilizing Ni ions from the crystallization conditions. HjGH61B has an immunoglobulin-like β-sandwich fold, and very atypically if it were a true GH, lacks a clear substrate binding groove and an appropriately positioned and exposed active site carboxylate pair, which is, with few exceptions, ubiquitous in GHs mechanisms. The authors concluded that based on the structure this was an unlikely GH. Most interestingly, a clear metal binding site was revealed in each of the two molecules in the asymmetric unit, and the coordinated metal ion was assigned as Ni due to the presence of this metal in the crystallization conditions. The metal protein ligands were the N-terminal His as well as an additional His and a Tyr, all noted to be conserved in GH61 sequences and thus of potential functional importance. However the nature or indeed a presence of the native metal could not be established in solution despite attempts to do so by particle-induced X-ray emission. Furthermore no link to a function or activity could at this point be made.

The structure of GH61E from *Thielavia terrestris* (TtGH61E), determined by Multiple Isomorphous Replacement, was preliminarly presented in 2008 [12] and published in 2010 [14]. *Thielavia terrestris* is a thermophilic cellulose degrading ascomycete, which when cultured on cellulose secretes a number of known cellulases and hemicellulases, but also at least six GH61 proteins, making up about 10% of total soluble protein [14]. TtGH61E has only 29% sequence identity with HjGH61B, yet shares many significant structural features, notably the divalent metal binding site. As well as structure determination of TtGH61E, Harris and coworkers present in [14] the first GH61 activity assay, previously disclosed in the patent literature [15]. The assay measured GH61 activity in terms of their boosting effect on the activity of conventional hydrolytic cellulases. It should be noted that cellulase boosting activities, which now can be assigned to GH61 enzymes, were reported already in conferences well ahead of this time, for example for a fungal extract at the 2003 MIE Bioforum [16]. The establishment of an assay, although as it turned out later a rather indirect one, was instrumental to the major breakthrough of this publication, the establishment of a firm link between the found metal binding site and cellulose degradation. By combining knowledge of the structure with an assay, the requirement of the metal for boosting activity was demonstrated by structure-guided site directed mutagenesis, showing loss of this activity in variants where the metal binding residues were removed. The His1 to Asn and His68 to Ala mutants were completely inactive, while a Tyr153 to Phe mutant had reduced activity. Mutation of Gln151 (H-bonding to Tyr153) to a variety of residues reduced activity even more severely.

In the HjCel61B and TtGH61E structures, the divalent metals were assigned purely on the basis of their presence in the crystallization mixture, following the normally good crystallographic practice of modeling only chemical entities that are known or can be proven by other means to be present in the mixture. Thus the identity of the metal binding site in its native environment remained unclear. The demonstration that GH61 could boost the activity of hydrolytic cellulases established a biological justification for the coexistence and co-expression of GH61 with the more conventional cellulose-degrading enzymes and underpinned the biotechnological potential of GH61. It is noteworthy that these first structural publications included authors from two large world enzyme producers, Novozymes A/S and Genencor. As can be seen from Figure 1b, after the publication of the TtGH61E article the literature on GH61 was significantly boosted.

**Figure 2:**
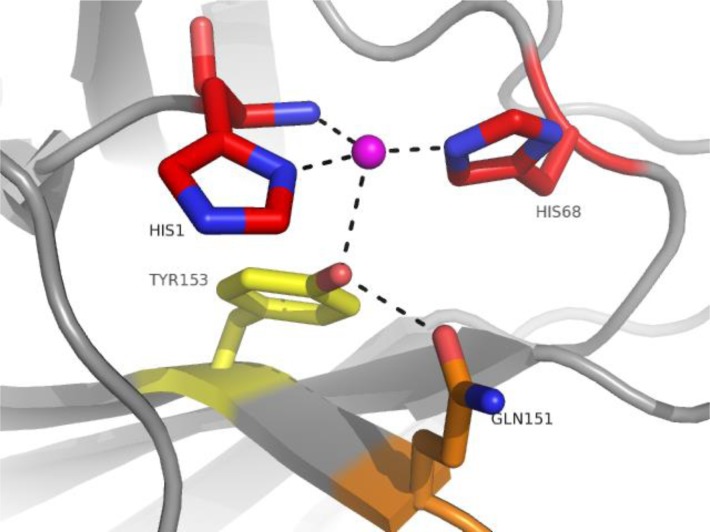
Metal binding site of TtGH61E (PDB code 3EJA, chain A) highlighting the mutated residues [14]. In red residues whose replacement resulted in complete abolishment of activity. In orange Gln151 whose replacement resulted in complete abolishment or severe impairment of activity (depending on the replacement residue). In yellow Tyr153 whose replacement resulted in impairment of activity.

Another very important link emerging from these first two structures was the structural similarity to chitin binding protein CBP21 from *Serratia marcescens* [17] at that point thought to non-enzymatically disrupt chitin and classified as a carbohydrate binding module belonging to family 33 (CBM33). Not only the overall structure of CBP21 was similar to the two GH61 structures, but CBP21 had also a very similar arrangement of residues as in the identified metal binding site, although no divalent metal was modeled in this structure (a sodium ion is though modeled at this site in one of the molecules in the asymmetric unit, see PDB code 2BEM). Furthermore mutagenesis of the conserved non-terminal His at this site was shown to affect the boosting effect of CBP21 on chitinase C [18]. There is however an important difference at the metal binding site since CBP21 has a conserved Phe instead of Tyr, while a Tyr to Phe substitution is detrimental to the activity of TtGH61E.

Given the functional and structural similarities and their complementary phylogenetic distribution (GH61 are predominantly fungal – see also the phylogenetic analyses in [14] and [19] - while CBM33 are predominantly bacterial and viral), it was already suspected at this time that the two families maybe distantly evolutionarily related. More details on the CBM33 family can be found in a recent publication which reviews the two families’ (CBM33 and GH61) biotechnological potential [20].

Although this review strictly focuses on GH61, the progress in the understanding of the two families has been so linked that CBM33 cannot be completely ignored. A key paper in the understanding of the mechanism showed that CBM33 proteins oxidatively degrade crystalline chitin [21] producing a mixture of oxidized and unoxidized even-numbered chitooligosaccharides, preferentially of high DP. One of the oxygens in the oxidized products came from water while the other was contributed by molecular oxygen. Furthermore this publication established that a divalent metal ion was necessary for oxidative function although the nature of the metal requirement was not firmly established. It was later shown that CBM33s are not exclusive to chitin degradation but are also able to degrade cellulose, since CelS2 from *S. coelicolor* A3(2) was shown to degrade crystalline cellulose with production of oxidized (aldonic acids) and unoxidized products, again with dominance of even-numbered DP [22]. Similarly to previous work, divalent metal ions were shown to be necessary, but no clear preference could be shown.

Not long after researchers began to report oxidative activity also for GH61 proteins, resulting in a mixture of non-oxidized and oxidized cellodextrins after crystalline cellulose degradation [4, 23, 5]. In [4], it was clearly shown that at pH 5 a GH61 protein from *Thermoascus aurantiacus*, TaGH61A, is highly selective for binding of Cu^2+^ ions and that the Cu-loaded TaGH61A oxidatively degrades crystalline cellulose in the presence of small molecule redox active agents such as gallate and ascorbate. Electron paramagnetic resonance spectroscopy showed clearly a signal for Cu(II) similar to the one observed in type II copper oxygenases. More or less at the same time [24], it was also shown that the combination of GH61 and cellobiose dehydrogenases (CDH) from same or different organisms could oxidatively degrade highly crystalline cellulose without added small molecule reductants. Later in 2011, reports in [5] and [6] confirmed that GH61 are cellulose degrading Cu metalloenzymes. In [5] the important role of cellobiose dehydrogenases (CDH) in cellulose degradation by GH61 was underpinned by genetic and biochemical experiments, and it was suggested that in nature reduced CDHs may reduce Cu(II) to Cu(I) in the catalytic mechanism of GH61s. Furthermore it was suggested that polysaccharide monooxygenases, as GH61 are referred to in this publication, can be of two types depending on whether oxidation is introduced on one side or the other of the broken glycosidic bond. The mechanisms of the type 1 and type 2 GH61s have been investigated in more detail in [25] and by isotope labelling GH61s of type 1 were shown to incorporate one oxygen atom from molecular oxygen into the product and therefore to be monooxygenases. Two crystal structures for *Neurospora crassa* GH61s have recently been published [26], where the authors claim to have isolated dioxygen species, which however are difficult to unequivocally establish purely by crystallography, despite high resolution and careful refinement. As a final remark, after the very recent elucidation of the chitinolytic system of *Enterococcus faecalis* V583 and structure determination of a new CBM33 enzyme, Cu has after some dispute been recognized to be the active metal also for these enzymes [27]. Thus there seems to be general consensus that both GH61 and CBM33 are Cu dependent monooxygenases.

Structural biology has thus made an essential contribution to the understanding of the molecular mechanism of GH61 function, especially by the discovery of the similarity between GH61 and CBM33 and the metal binding site, its functional importance (by guiding mutagenesis) and its clarification as a Cu(II) binding site. The structures known so far for GH61 and some of their features are reviewed below.

## Known structures of GH61 family members

**Figure 3:**
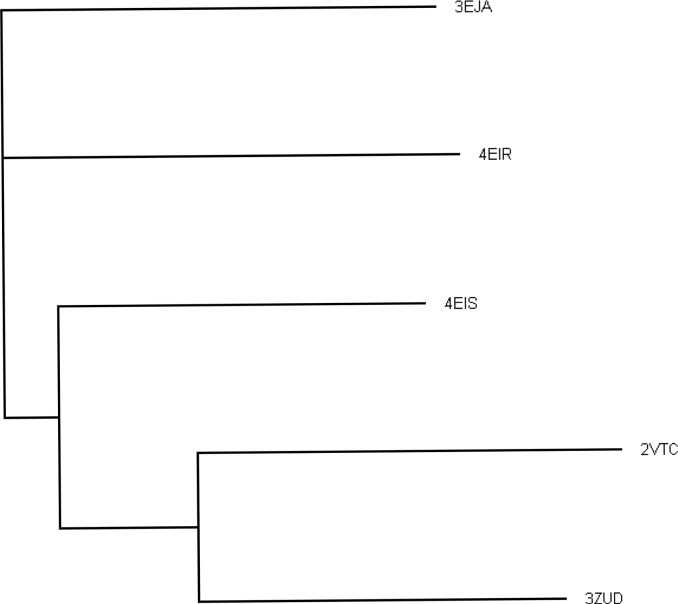
Dendrogram (produced by near joining method in ClustalW2 [29] and displayed with Treeview [30]) graphically showing the similarities between the proteins of known structure, indicated by their PDB codes (the proteins corresponding to each PDB code can be found in Table 1). The multiple structural alignments were carried out with the server version of Mammoth-Mult [31].

**Figure 4:**
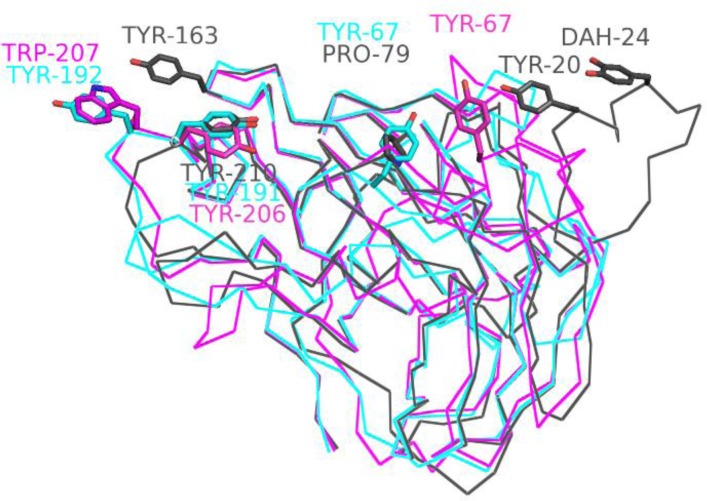
Residues contributing to the flat potential substrate binding surface of TtGH61E (cyan), NcPMO2 (magenta) and NcPMO3 (grey). DAH is the hydroxylated form of Tyr24.

To date, five structures of GH61 proteins have been determined, all by X-ray crystallography. Table 1 summarizes some of the characteristics of the structures, while Table 2 summarizes the sequence and structural similarity between them. Two of the structures (TtGH61E and HjGH61B) have already been discussed in some detail above and are in fact the two most dissimilar (Dali-Lite [28] aligns 200 residues with 1.9 Å Cα rmsd and 29% structure based sequence identity). Three additional structures have since been determined [4, 26]. The dendrogram in Figure 3 illustrates graphically the relationship between the different proteins. TtGH61E (code 3EJA) and NcPMO2 (code 4EIR) are distant from each other and the other sequences, while NcPMO3 (code 4EIS), HjGH61A (code 2VTC) and TaGH61A (code 3ZUD) form a more closely related group. This is also highlighted in Figure 4, where differences in loop structures as well as presumed functional residues are clearly visible between TtGH61E, NcPMO2 and NcPMO3.

**Table 1:** Overview of known structures. The Tyr conserved at the aromatic surface in all structures is in bold.

Abbreviation	Organism	PDB code(s)	Resolution (Å)	Associated with CBM1	‘Flat’ surface aromatics	Ref
TtGH61E	*Thielavia terrestris*	3EII 3EJA	2.25 1.90	No	Tyr67, **Tyr191**, Tyr192	[14]
NcPMO2	*Neurospora crassa*	4EIR	1.10	No	Tyr67, **Tyr206**, Trp207	[26]
TaGH61A	*Thermoascus aurantiacus*	3ZUD 2YET	1.25 1.50	No	Tyr24, **Tyr212**	[4]
HjGH61B	*Hypocrea jecorina (Trichoderma reesei)*	2VTC	1.60	No	Tyr23, **Tyr212**	[13]
NcPMO3	*Neurospora crassa*	4EIS	1.37	No	Tyr20, Tyr24, Tyr163, **Tyr210**	[26]

**Table 2:** Structure similarity from Dali-Lite [28] (number of aligned residues, Cα rmsd and structure-based sequence identity are shown).

	TaGH61A (3ZUD, A)	TtGH61E (3EJA, A)	HjGH61B (2VTC, A)	NcPMO2 (4EIR, A)	NcPMO3 (4EIS, A)
**TaGH61A**	-	201 res	220 res	205 res	218 res
**(3ZUD, A)**		1.7 Å	1.3 Å	1.7 Å	1.5 Å
		33%	47%	31%	41%
	**TtGH61E**	-	200 res	198 res	199 res
	**(3EJA, A)**		1.9 Å	1.7 Å	1.5 Å
			29%	39%	42%
		**HjGH61B**	-	205 res	216 res
		**(2VTC, A)**		1.8 Å	1.7 Å
				28%	37%
			**NcPMO2**	-	204
			**(4EIR, A)**		1.7 Å
					42%
				**NcPMO3** **(4EIS, A)**	-

## Association of GH61 catalytic domain with Carbohydrate Binding Modules (CBMs)

As noted also in the early reports on GH61 gene cloning and sequences, these catalytic domains are often associated with CBMs [2], and in particular with CBM1. In [14] about 20% of GH61 sequences were estimated to be associated with a C-terminal CBM1 (an N-terminal CBM would interfere with the N-terminal His metal-binding function). CBM1s are A-type CBM, typically presenting a flat surface which binds to a crystalline polysaccharide, for CBM1 usually cellulose [2]. A recent search using the Cazymes Analysis Toolkit (CAT [32]) shows that 37 out of 143 (26%) of GH61s in CAT are associated with CBM1, indicating that the estimate in [14] is holding up as new sequences come into CAZY. It seems that in organisms having multiple GH61 genes some are associated with a CBM1 and some are not, for example in *Heterbasidion irregulare*, three out of ten GH61 genes have an associated CBM1 sequence [33]. Two of the CAZY entries are interestingly associated with CBM18s, which are typically chitin fragment binding. This could perhaps suggest that like some CBM33s can degrade cellulose, some GH61 could be involved in chitin degradation. CBM18s are however normally considered type C CBMs, binding small fragments rather than crystalline polysaccharides. Interestingly, the CBM33 CelS2 protein shown to be active on cellulose [22] has a CBM2 associated with it (CBM2s are A-type binders usually binding to cellulose, but also to chitin or xylan).

None of the structurally characterized GH61 proteins has naturally a CBM attached. This is not surprising as successful crystallization is strongly biased towards single domain, compact proteins.

However for the TtGH61E structure [14], a CBM1-like feature within the catalytic domain was noted, three Tyr forming a flat surface and arranged similarly as the three Tyr in the structure of the CBM1 of *T. reesei* Cellobiohydrolase I [34]. For TtGH61E, crystalline cellulose binding activity has been qualitatively shown experimentally [35] and it has been shown that substitution of one of the aromatics (Tyr192) to Ala reduces activity [14]. Although this CBM1-like feature is not generally conserved, all of the structures of GH61 determined so far have a ‘flat’ face, to which two or more aromatic residues contribute (see Table 1 and Figure 4 for illustration of three of the most diverse structures) which could be involved in crystalline substrate binding. Tyr191 of TtGH61E has an equivalent Tyr in all other structures determined (in bold in Table 1). A structural equivalent of Tyr192 is present as Trp207 in NcPMO2, otherwise the loops differ here between the structures. The third Tyr forming the flat surface in TtGH61E is often but not always a Pro in other structures (HjGH61A, TaGH61A and NcPMO3). NcPMO3 has, together with the Tyr conserved in all structures, additional aromatics Tyr20, Tyr24 and Tyr163 which form a flat aromatic surface, while NcPMO2 has a Tyr67 (which is not equivalent of Tyr67 in TtGH61E). Differences in the putative binding surfaces of GH61 have also been discussed in [26] and a flat binding surface has also been described for CBM33 [27].

## The metal binding site

As stated above, there is currently reasonable consensus that the active metal in GH61 proteins is copper, however it seems that GH61 (and CBM33) can be sometimes demetallated/substituted for other metals during overexpression and purification despite the high affinities for Cu at active pHs. One possibility is that this is mediated by changes in pH during purification. In Table 3, the distances to protein residues for the structures where metal at the active site could be positively identified as Cu (3ZUD, 4EIS, 4EIR) are shown and are very typical and very similar in the three proteins. The Cu(II) is in tetragonal coordination geometry in all structures reported. The protein ligands are highly conserved in GH61 sequences and in all the structures reported so far.

**Table 3:** Cu-protein distances in TaGH61A (3ZUD), NcPMO2 (4EIR) and NcPMO3 (4EIS). For 3ZUD the distances are to the main conformation of the Cu atom. In 4EIR and 4EIS there are two molecules per asymmetric unit, hence two distances are given.

	N-terminal His N	N-terminal His ND1	His NE2	Tyr OH
3ZUD	2.2 Å	1.9 Å	2.0 Å	2.9 Å
4EIR	2.2/2.2 Å	1.9/1.9 Å	2.0/2.0 Å	2.8/2.8 Å
4EIS	2.3/2.3 Å	1.9/1.9 Å	2.1/2.1 Å	2.7/2.8 Å

**Figure 5:**
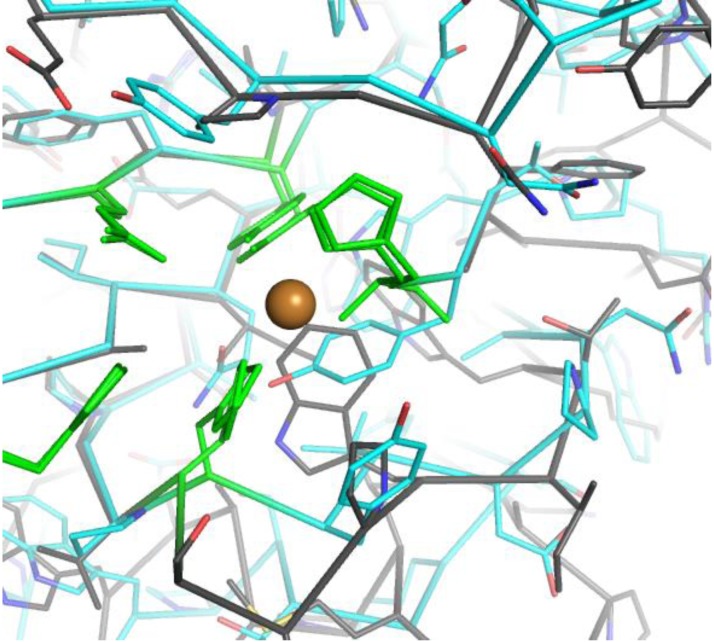
Structural diversity around the Cu binding site. TtGH61E is shown in cyan, while NcPMO3 in grey. The conserved Cu-binding residues and additional His and Gln are in green.

The special spatial arrangement of the two coordinating His (one coordinating both with the N-terminus and its side chain) has been named ‘histidine brace’ [4], and a similar arrangement has also been observed in copper methane monooxygenases, which however differ by having two rather than one Cu atoms at a distance of 2.7 Å from each other, as confirmed by a recent 2.68 Å resolution structure [36]. Other conserved residues around the metal binding site are the Gln hydrogen bonding to the conserved Tyr and a third His residue (His164 in TaGH61A), the function of which is yet unknown. These are interestingly not the same in CBM33. In CBP21 an Asn (185) and an Asp (182) are equivalent to the GH61 Gln and His, respectively, and the two residues are highly conserved as Asn and Asp in the family. Only mutation of Asp182 to Ala affected the combined activity of CBP21 and chitinase C, while mutation of Asn185 had little effect [18]. This correlates well to the fact that the equivalent of the conserved Tyr is in CBM33 a Phe, which could not form a hydrogen bond with a polar residue. Aside from the residues directly coordinating the ligands and the additional Gln and His, there is considerable diversity in the residues immediately surrounding the Cu(II) site among the different GH61 proteins, as illustrated in Figure 5 by the structures of TtGH61E and NcPMO3. This diversity may prove important in modulating the activities of different GH61.

The N-terminal His plays a special role in coordinating the metal, as it provides two ligands, a main chain and a side chain nitrogen. In [4] it was first recognized that this N-terminal His is a site of unusual post-translational modification, a methylation at Nɛ2. This was supported by crystallographic analysis and mass spectrometry for *T. aurantiacus*. Reanalysis of previously reported structures, and modelling of methylation in all subsequently reported structures suggests that this may be a feature of all active GH61 proteins. No evidence of such a modification has been presented for the bacterial CBM33s.

Intriguingly, although the N-terminal His is extremely well conserved in GH61, some GH61 members have an Arg at this position, for example HiGH61G from *Heterobasidion irregulare* [33], which also lacks the other Cu-coordinating His. Even more intriguingly, it seems that some of these proteins (including HiGH61G) are upregulated when fungi are grown on lignocellulosic substrates, as well as GH61s having an integral metal binding site (as judged by sequence). This observation opens the possibility that some GH61 may have additional and metal-independent roles in cellulose degradation.

## Outlook

One of the areas of interest in terms of exploiting the biotechnological potential is of course discovery of novel GH61 enzymes. In this sense it seems that genome mining and generally ‘omics’ analyses in lignocellulose degrading organisms may prove to be a very fruitful strategy for GH61 as well as other plant cell wall degrading enzymes. Especially white-rot fungi such as *Phanerochaete chrysosporium* [19], thermophilic biomass degrading fungi where the GH61 family is largely expanded – eg. *Thielavia terrestris*, boasting 18 GH61 genes compared to 3 in *T. reesei* [37] - and plant pathogens [38] have been subject of great attention. In the last few years, upregulation of some, but not all, GH61s upon growth on cellulosic substrates has been observed in transcriptome and secretome analyses of *P. chrysosporium* [19], transcriptome analysis of *Phanerochaete carnosa* [39], proteomic analysis of of *Aspergillus nidulans* growing on sorghum stover [40], and qRT-PCR studies on GH61 of the pathogen *Heterobasidion irregulare* [33], where HiGH61H showed a rather spectacular 17,000 fold increase on spruce heartwood. Since some GH61 genes/proteins are not upregulated by growth on cellulosic substrates, these may have different substrate specificities, which are yet to be explored.

The interplay between CDH and GH61 needs to be explored further. Co-induction of CDH and GH61 upon growth on cellulosic substrates has been reported in several large scale studies and organisms, among others *P. chrysosporium* [19], *Aspergillus nidulans* [40] and *Thielavia terrestris* [24, 23]. It has also been suggested that lignin may be sufficient as a reductant, as no addition of small molecules reductants is needed when lignin is used [41, 42].

Although oxidative enzymes like GH61 and CBM33 act in synergy with glycoside hydrolases, the final oxidised products of the reaction can pose a limit to the final yields that can be obtained. Gluconic acid, which can be a significant proportion of overall reaction products from commercial enzyme preparations [41], is a known inhibitor [43] of β-glucosidase and more inhibitory to β -glucosidase activity than glucose in realistic reaction mixtures [41]. Furthermore it is not fermentable by *S. cerevisiae*. Cellobionic acid is also a worse substrate than cellobiose for β-glucosidases [41]. A better understanding of the interplay of different components is necessary, to achieve as high as possible yields of conversion.

Many challenges remain also on the fundamental understanding of GH61 action, their interaction with substrate and the electron transfer pathways. Crystallography is expected to continue contributing to the elucidation of GH61 detailed function and diversity. However a true molecular understanding will not come from structural biology alone, but requires combined efforts involving experts from different fields including phylogenetics, transcriptomics and bioinorganic chemistry.
